# Supplementary material for: Development and Verification of a Deep Learning Algorithm to Evaluate Small-Bowel Preparation Quality
Source: Diagnostics (Basel). 2021 Jun 20;11(6):1127. doi: 10.3390/diagnostics11061127 (PMC8234509; doi:10.3390/diagnostics11061127)
Supplement: Supplementary file 1 [file diagnostics-11-01127-s001.zip › diagnostics-1202796-supplementary.pptx]

## Slide 1
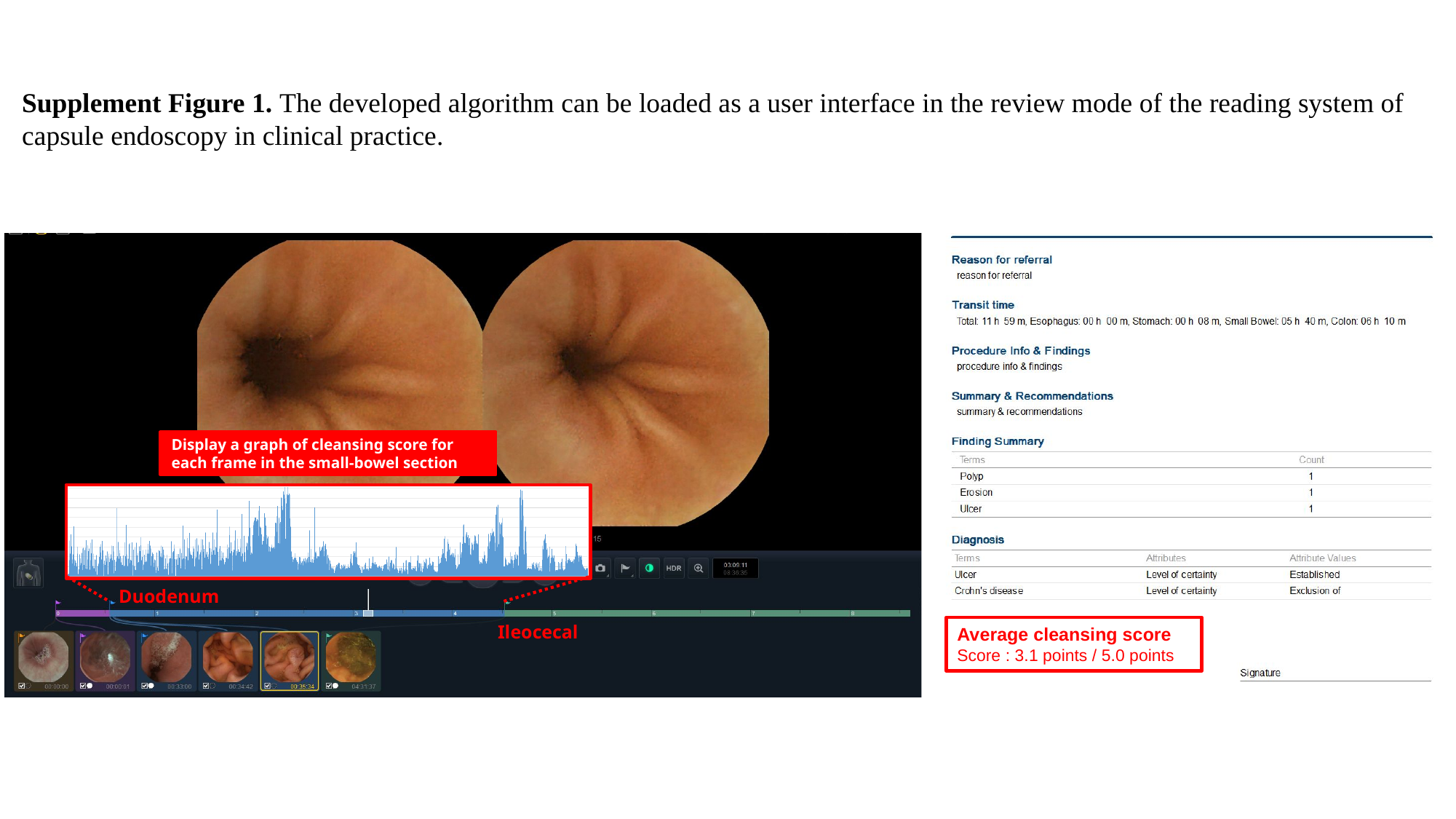

Supplement Figure 1. The developed algorithm can be loaded as a user interface in the review mode of the reading system of capsule endoscopy in clinical practice.
Display a graph of cleansing score for each frame in the small-bowel section
Duodenum
Ileocecal
Average cleansing score
Score : 3.1 points / 5.0 points
